# 

**Published:** 1888-12-01

**Authors:** 


### THE STUDENT'S COLUMN.

### UNIVERSITY OF LONDON, 1888.
### M.B. EXAMINATION.

Examiners:

*Medicine:* William Cayley, Esq., M.D., and Prof. F. T. Roberts, M.D., B.Sc.
*Surgery:* W. Morrant Baker, Esq., and Prof. Christopher Heath.
*Obstetric Medicine:* F. H. Champneys, Esq., M.D., M.A., and Prof. John Williams, M.D.
*Forensic Medicine:* Prof. G. V. Poore, M.D., B.S., and Thomas Stevenson, Esq., M.D.

The following is the pass list, arranged as usual, in two divisions:—

First Division.

Abram, John Hill, University Colleges, Liverpool and London.
Alcock, Samuel King, St. Bartholomew's Hospital.
Ashe, Evelyn Oliver, London Hospital.
Ashworth, Percy, B.Sc., Owens College and Manchester Royal Infirmary.
Bays, James Thomas, St. Mary's Hospital.
Bird, Robert, St. Bartholomew's Hospital.
Bradford, John Rose, D.Sc., University College.
Brock, Ernest Henry, Guy's Hospital.
Carter, Weldon Cragg, University College.
Clarke, James Jackson, St. Mary's Hospital.
Crook, Herbert Evelyn, Guy's Hospital.
Dean, Henry Percy, B.Sc., University College.
Fernando, Hilarion M., B.Sc., University College.
Firth, John Lacy, University College.
Francis, Alfred George, St. Bartholomew's Hospital.
Gould, John Edwin, University College.
Kanthack, A. A., B.A., B.Sc., Liverpool Royal Infirmary and St. Barth's.
Leech, Priestley, Owens College.
Lyndon, Arnold, St. Bartholomew's Hospital.
Macevoy, Henry John, B.Sc., St. Thomas's Hospital.
Maillard, William Job, Guy's Hospital.
Mair, Ludovic W. Darra, St. Bartholomew's Hospital.
May, William Page, B.Sc., University College.
O'Reilly, George H., Northampton General Infirmary and King's Coll.
Parkin, Alfred, Guy's Hospital.
Parkinson, John Porter, University College.
Pierce, Bedford, St. Bartholomew's Hospital.
Ransom, William Bramwell, B.Sc., University College.
Roberts, John Lloyd, B.A., B.Sc., Guy's Hospital.
Roper, Harold Kennaway, Guy's Hospital.
Sansom, Harry Arthur, St. Thomas's Hospital.
Solly, Reginald Vaughan, St. Thomas's Hospital.
Starling, Ernest Henry, Guy's Hospital.
Swayne, Walter Carless, Bristol Medical School and Guy's Hospital.
Tonking, John Herbert, St. Thomas's Hospital.
Wheeler, James Atkin, Guy's Hospital.
White, Gilbert Benjamin Mower, University College.
Wilkie, John, B.Sc., St. Bartholomew's and Brompton Consumption Hosp.
Williams, Herbert, St. Bartholomew's Hospital.
Willoughby, William George, St. Bartholomew's Hospital.

Second Division.

Blaxall, Frank Richardson, University College.
Brown, Arthur Thomas, Guy's Hospital.
Brown, Edward Vipont, St. Bartholomew's Hospital.
Canney, Henry Edward Leigh, University College.
Cuff, Herbert Edmund, Guy's Hospital.
Duncan, Horace, St. Thomas's Hospital and Cambridge.
Edge, Frederick, B.Sc., Owens College and Manchester Royal Infirmary.
Elphick, Harry William, University College.
Grayling, Arthur, St. George's Hospital.
Hastings, Edwin Birchall, University College.
Hicks, John Sydney, London Hospital.
Joseph, A. H., Medical School and Royal Infirmary, Bristol, and King's Coll.
Lang, George Herbert, University College and Manchester Royal Infirmary.
Little, Arthur Nicholas, Bristol Medical School.
Macdonald, Isabella M., London School Medical and Royal Free Hospital.
Melland, Brian, Owens College and Manchester Royal Infirmary.
Moss, Enoch, Guy's Hospital.
O'Brien, Patrick Moriarty, University College, Liverpool.
Oliver, Charles Pye, Charing Cross Hospital.
Randall, Philip Nicholas, Guy's Hospital.
Smith, Guy Bellingham, Guy's Hospital.
Smith, Thomas Wilson, Guy's Hospital.
Sutherland, G. W., B.A.Syd., Univ. College London and Univ. Edinburgh.
Symonds, Henry, St. Bartholomew's Hospital.
Taylor, Charles Henry, King's College.
Tidey, Stuart Alexander, St. Mary's Hospital.
Tresidder, William Elliot, Guy's Hospital.
Tunnicliffe, Francis Whittaker, St. Bartholomew's Hospital.
Webb, Helen, London School Medical and Royal Free Hospital.
Wills, Ernest, University College.
Wilson, Charles, London Hospital.

**Notice to Advertisers and Subscribers.**

All Advertisements, Orders for Copies of The Hospital, and Business Communications, should be addressed to The Publisher, *not to the Editor*, The Hospital, 38, Old Jewry, E.C.

**To Residents, Secretaries, and Matrons.**

The Editor will be glad to have the Regulations and each Report as issued by Asylums, Hospitals, Convalescent and Nursing Institutions, as well as those of other Charities, and an early copy in duplicate of all Appeals and Festival Notices, etc., addressed to *The Editor, The Lodge, Porchester Square, London, W.* Any superintendent, secretary, matron, or other chief official who regularly sends such information may be registered, on application to the Publisher, to receive free of cost a cover for binding The Hospital in half-yearly volumes.

### Scraps and Gleanings.

Hospital Sunday at Burton brought in £404.

The *Medical Press* says that leprosy is increasing in Russia.

There are 102 women medical students now at the Swiss universities.

Mr. Gifford has joined Father Damian among the lepers of Molokai.

There are two working men on the board of the Derbyshire Infirmary.

Dr. Lloyd Owen is appointed first Middlemore Lecturer at Birmingham.

The subscriptions received at the Lincoln Dispensary Ball amounted to £122.

All the schools are closed at Brixham, owing to an outbreak of scarlet fever.

The Metropolitan Asylums Board has no small pox cases in its hospitals just now.

Dr. Valentine, of Earlestown, has been presented with a microscope by his ambulance pupils.

It is proposed to hold a bazaar in aid of Bootle Borough Hospital in April next. £1,000 are wanted.

The Bradshawe Lecture will be delivered by Mr. Jonathan Hutchinson on December 6th at four p.m.

Mansfield Hospital needs a new surgical ward, which cannot be commenced till £800 is subscribed.

The Morton Lecture on "Cancer" will be delivered by Sir Spencer Wells on November 29th at four p.m.

It is proposed to amalgamate the Mickleover and Matlock Bank Convalescent Homes in Derbyshire.

A Concert was given last week by the Magpie Minstrels, in aid of the Hampstead Nursing Association.

The National Temperance League have awarded three prizes for essays on the advantages of Temperance.

The Manchester Cremation Society believes that a crematory for the Midlands will be built early next year.

On November 10th, a presentation was made to Dr. Ord in recognition of his services as Dean of St. Thomas's.

St. John's Hospital, Twickenham, has secured the conditional £200 offered by Mr. Cunard and Mr. Twining.

Lady Grant Duff gave an interesting account of her personal experience of Zenana work, at York House last week.

The sermon at St. Patrick's Cathedral, Dublin, on Hospital Sunday was preached by the Ven. Canon J. G. Scott.

The annual Dispensary Ball took place at Grimsby on November 8th, the Lady Patroness being Mrs. F. H. Kerans.

A writer to the *Globe* declares that doctors and nurses often carry infection to their patients. No statistics are given.

Two ladies have passed the first examination in medicine, and two the second examination, at Dublin University.

A Zenana Hospital was opened at Quetta, Beloochistan, on November 2nd, thanks to Lady Dufferin's movement.

Many of the lodging-house keepers at Blackpool object to Mrs. Reaney starting a Convalescent Home in their midst.

The North-East Lancashire Needlework Association keeps Blackburn Infirmary supplied with a large quantity of clothing.

Dr. Day informs the Brentford Board of Guardians that the beer supplied to the infirmary is so bad as to make the patients ill.

St. Albans Hospital has a patient with several broken ribs, whose injuries are the result of a prize fight on Colney Heath.

Dr. Metzger, the famous *masseur* of Amsterdam, has decided to transfer his domicile to Wiesbaden at the beginning of next year.

A Sydenham chemist has been fined for supplying a girl with laudanum in a vessel not marked with the seller's name and address.

The profits arising from the sale of Mr. Rodd's "Life of the Emperor Frederick" are to be given to the London Throat Hospital.

A concert was given at Newtown last week, in aid of the Montgomeryshire Infirmary. Some of the songs were sung in Welsh.

On November 19th Miss M A. Chreeman delivered a lecture at the Richmond Athenæum on "The Physical Culture of Women."

Miss Dodds, Dean of the Hygienic College of St. Louis, has been appointed President of the Hygienic Convention of the United States.

The *Englishwoman's Review* for November 15th contains an interesting article on the "Present Condition and Future Prospects of Medical Women in India."

A Demonstration of North London amalgamated temperance societies took place last week in aid of the building fund of the Great Northern Central Hospital.

Several cheques are being received at the Dublin Mercers' Hospital, from those who think it has been unjustly excluded from participating in the Sunday Fund.

The *Oddfellows' Magazine* for November speaks very highly of the proposed Convalescent Home for Members of Friendly Societies in the County of Durham.

Christoper Crayon has contributed an article to the *Christian World* on "A Seaside Hospital." It deals with the Friedenfels Home for Consumptives, Hastings.

The Board of Guardians of Cahiceveen have passed a resolution of sympathy with Mrs. Spotswood on the death of her husband, for 45 years medical officer of the district.

Dr. C. H. Willey, of Sheffield, has, on the occasion of his leaving the Borough Hospital, been presented by the staff of the institution with a handsome case of cutlery.

A concert was given at Princes' Hall on November 16th, in aid of the Princess Frederica's Convalescent Home. Miss Liza Lehmann sang well, and was heartily applauded.

Dr. Anderson has written to the *Presbyterian Messenger* complaining of the difficulties the Chinese authorities place in their way against securing a site for a new hospital at Taiwanfoo.

### Amusements and Relaxation.

### NEW PUZZLE PRIZE SCHEME.

COMMENCED OCTOBER 6th, ENDS DECEMBER 29th, 1888.

On October the 6th a New Prize Scheme was commenced in these columns.

Prizes will be awarded for the best Original Puzzles in Prose or Verse, relating to any of the *Social, Philanthropic, or Political Topics of the day*, and taking the form of Double Acrostics, Anagrams, Square Words, Decapitations, Transpositions, or any other Puzzles which the ingenuity of the competitors may suggest. (*Answers must accompany contributions.*)

*Readers* are requested to send in weekly the Number of Marks they consider each composition to be entitled to, three being the highest score for any one contribution.

Eight Prizes of Standard Books will be given, the choice of the book to be left to the winner.

First, Second, Third, Fourth, and Fifth Prizes for best Original Puzzles, of £1 1s., 15s., 10s. 6d., 7s. 6d., and 5s. each.

First, Second, and Third Prize for Solutions of 15s., 10s. 6d., and 5s. each.

N.B.—All contributions must reach the office, 38, Old Jewry, E.C., not later than the *First Post* on *Thursday* in each week.

### PUZZLES FOR SOLUTION.

**Seventh Week.**

No. 18. Charade.

Against the rocky coast my *first* is beating,
And sometimes borne upon the waves retreating;
My *next* transposed is a denial,
If on the battle field in pain you're roaming,
Or on a bed of sickness you are moaning;
My *whole* may ease you in your trial.

G. D.

No. 19. Diagonal Puzzle.

Read me crossways and you'll find
Two sisters here of different mind.
1. Near the Balkan my name you'll see,
2. How, what, why, when? stand for me.
3. We are young birds fierce and strong,
4. A brave deed done for right or wrong.
5. Very high talk from a very small mind,
6. You can see bits of me, in every kind—
7. Hard and clear, and dazzling bright,
Men for me will rob or fight.

Morico.

No. 20. Enigma.

Spurned as all useless by the legal mind,
I turn to doctors, and a welcome find,
'Tis *accent* only that assigns my lot,
'Mid these who prize or those who prize me not.

Lady Clara.

**Marks.**

| Names. | No. of Puzzles sent in Nov. 22. | No. of Marks Nov.22 | Total of Marks. | Names. | No. of Puzzles sent in Nov. 22. | No. of Marks Nov.22. | Total of Marks. |
|---|---|---|---|---|---|---|---|
| Patience .... | 3 | 6 | 38 | Hercules .. | 2 | 0 | 11 |
| Lady Clara.. | 3 | 7 | 43 | Gretta ...... | 3 | 6 | 28 |
| Tinie ...... | 3 | 6 | 36 | Dandy Dick | — | — | 5 |
| Bijou ...... | 1 | 2 | 17 | Boadicea .. | — | — | 7 |
| G. D. ...... | — | — | 15 | Nipper .... | 3 | 5 | 28 |
| Thanet .... | 2 | 5 | 27 | Morico .... | 3 | 9 | 27 |
| Daisy ..... | 3 | 3 | 14 | A. M. E..... | — | — | 3 |
| Salford .... | — | — | 6 | John Gilpin | — | — | 4 |

**Answers to Fifth Week Puzzles.**

No. 13. Double Acrostic.

T reasur E

E meral D

C hâtea U

H avo C

N ebul A

I nsigh T

C ad I

A lt O

L iche N

No. 14. Original Anagram.
"The Hospital Nursing Mirror."

No. 15. Original Charade. Band-age = Bandage.

**Marks for Solution of Puzzles.**

Total Marks.—Ruffles (2), 8; Daisy (1), 6; Lady Clara (1), 9; Reldas, (2), 8; Mater 4; Condorcet (1), 6; Tinie (2), 8; Gentian (2), 9; Marguerite, 5; Patience (1), 4; Gretta 3; Amicus (2), 6; Scrooge (2), 9; Smut, 2; Thanet (2), 12; Jenny Wren (1), 2; Lauriston (1).

*The figures in parentheses are Marks for the week ending Nov. 22.*

**Erratum**—For light I of No. 9 Triple Acrostic, for "Peri A sti C," read "Peir A sti C."

**Conditions.**

I. Every paper sent in must be clearly written, and one side only must be used. All competitors must mark at the head of their papers the number of their competition.

II. Each paper must be signed by the author with his or her real name and address. A *nom de plume* may be added if the writer does not desire to be referred to by us by his real name. In the case of all prizewinners, however, the real name and address will be published.

III. All communications relating to prize competitions should be addressed to "The Prize Editor, The Hospital, 38, Old Jewry, London, E.C." Competitors are requested not to include in letters, etc., to the Prize Editor, communications on matters of other business. All letters must be fully prepaid.

IV. The Prize Editor of The Hospital is the sole judge of the competitions, and his decision is in all cases final and without appeal.

V. The Prize Editor reserves the right to publish any paper sent in, whether it receives a prize or not. He does not hold himself responsible for any MSS. sent, nor can he undertake to return rejected competitions.

